# A systematic review on the integration of explainable artificial intelligence in intrusion detection systems to enhancing transparency and interpretability in cybersecurity

**DOI:** 10.3389/frai.2025.1526221

**Published:** 2025-01-28

**Authors:** Vincent Zibi Mohale, Ibidun Christiana Obagbuwa

**Affiliations:** Faculty of Natural and Applied Sciences, Department of Computer Science and Information Technology, Sol Plaatje University, Kimberley, South Africa

**Keywords:** intrusion detection systems, cyber threats, explainable artificial intelligence, systematic review, model explainability, model interpretability, machine learning

## Abstract

The rise of sophisticated cyber threats has spurred advancements in Intrusion Detection Systems (IDS), which are crucial for identifying and mitigating security breaches in real-time. Traditional IDS often rely on complex machine learning algorithms that lack transparency despite their high accuracy, creating a “black box” effect that can hinder the analysts’ understanding of their decision-making processes. Explainable Artificial Intelligence (XAI) offers a promising solution by providing interpretability and transparency, enabling security professionals to understand better, trust, and optimize IDS models. This paper presents a systematic review of the integration of XAI in IDS, focusing on enhancing transparency and interpretability in cybersecurity. Through a comprehensive analysis of recent studies, this review identifies commonly used XAI techniques, evaluates their effectiveness within IDS frameworks, and examines their benefits and limitations. Findings indicate that rule-based and tree-based XAI models are preferred for their interpretability, though trade-offs with detection accuracy remain challenging. Furthermore, the review highlights critical gaps in standardization and scalability, emphasizing the need for hybrid models and real-time explainability. The paper concludes with recommendations for future research directions, suggesting improvements in XAI techniques tailored for IDS, standardized evaluation metrics, and ethical frameworks prioritizing security and transparency. This review aims to inform researchers and practitioners about current trends and future opportunities in leveraging XAI to enhance IDS effectiveness, fostering a more transparent and resilient cybersecurity landscape.

## 1 Introduction

With the rise of digitalization, the frequency and sophistication of cyber threats have grown exponentially, affecting sectors such as finance, healthcare, and government. This surge in cyber-attacks, often perpetrated by advanced threat actors and using sophisticated methods like polymorphic malware, has created an urgent need for cybersecurity frameworks capable of rapid detection and response (Kotecha et al., 2022; Arrieta et al., 2020). Intrusion Detection Systems (IDS) have become essential components of cybersecurity infrastructure, designed to identify and mitigate potential threats in real-time by analysing network traffic and user behavior (Lundberg and Lee, 2017). However, the demand for real-time, adaptable, and accurate intrusion detection has led to the incorporation of advanced machine learning (ML) and artificial intelligence (AI) models into IDS (Barredo et al., 2020). While these models improve detection accuracy, they often function as “black boxes,” offering high performance at the expense of interpretability (Adadi and Berrada, 2018; Lundberg and Lee, 2017).

The lack of transparency in traditional, AI-enhanced IDS presents a critical limitation. Security analysts must understand the reasoning behind each detection decision to verify, refine, and optimize model outputs, especially when the stakes are high, as in sensitive environments like financial and governmental networks (Arrieta et al., 2020). Uninterpretable IDS can lead to challenges such as high false-positive rates and difficulty responding to complex threats due to limited insight into model decision-making. This lack of accountability and interpretability is problematic for IDS applications, as effective cybersecurity frameworks require models that not only detect but also justify their decisions (Kotecha et al., 2022). Explainable Artificial Intelligence (XAI) has emerged as a powerful approach to address these transparency challenges in IDS. XAI techniques, such as SHAP (Shapley Additive Explanations), LIME (Local Interpretable Model-agnostic Explanations), and rule-based models, aim to make AI model predictions more interpretable, offering insights into the decision-making process (Tjoa and Guan, 2020; Lipton, 2018). These techniques allow security analysts to see how features contribute to classifying network activity as normal or suspicious, facilitating a more trustworthy and actionable IDS. For example, SHAP has been applied successfully in anomaly detection within IDS, helping analysts understand why certain traffic is flagged as malicious (Doshi-Velez and Kim, 2017; Ras et al., 2018). LIME provides local explanations for individual predictions, which is particularly useful for auditing specific detection decisions within IDS (Yang et al., 2021).

Despite the benefits, the application of XAI in IDS is still in its nascent stages, and several challenges remain. One major limitation is the computational overhead introduced by XAI techniques, as models such as SHAP and LIME are computationally intensive and may hinder real-time detection capabilities in high-speed environments (Adadi and Berrada, 2018). Additionally, there are trade-offs between interpretability and accuracy; simpler, interpretable models like decision trees may lack the precision of complex, “black box” neural networks in detecting nuanced threats (Samek et al., 2017; Barredo et al., 2020). Finally, privacy and security concerns arise as XAI models may expose sensitive patterns or features, potentially violating user privacy in certain contexts (Miller, 2019).

This systematic review addresses these gaps by analyzing recent research on integrating XAI techniques within IDS. Specifically, it investigates:

1. **Types of XAI Techniques**: Reviewing rule-based, SHAP, LIME, and hybrid models, focusing on their strengths and weaknesses when applied to IDS (Barredo et al., 2020).
2. **Challenges and Trade-offs**: Examining issues like interpretability versus accuracy, computational overhead, and real-time performance, which are critical considerations in high-stakes cybersecurity environments (Aldahdooh et al., 2021).
3. **Future Directions**: Exploring the potential for hybrid models and real-time XAI, suggesting ways to enhance the usability and reliability of explainable IDS for practical deployment in cybersecurity (Kotecha et al., 2022).

### 1.1 Additional contributions

As a key addition to the field, this review introduces a conceptual framework for integrating XAI into IDS. This framework provides practical guidelines for selecting XAI techniques based on specific operational requirements, such as real-time performance, regulatory compliance, and resource constraints. By offering this framework, the review not only summarizes the current state of the field but also provides actionable insights for researchers and practitioners aiming to deploy XAI-enhanced IDS in dynamic cybersecurity environments.

## 2 Literature review

The integration of Explainable Artificial Intelligence (XAI) into Intrusion Detection Systems (IDS) represents a pivotal advancement in cybersecurity, addressing the critical need for transparency and interpretability in AI-driven threat detection mechanisms. By bridging the gap between complex machine learning models and actionable insights, XAI enhances the usability and trustworthiness of IDS in high-stakes environments, such as finance, healthcare, and critical infrastructure (Arrieta et al., 2020; Barredo et al., 2020). This section provides a comprehensive review of contemporary XAI approaches applied to IDS, categorized into four key subtopics.

### 2.1 Importance of XAI in transparency and interpretability

The integration of XAI into IDS is critical for ensuring that cybersecurity systems are not only highly accurate but also capable of providing explanations that human analysts can readily comprehend and act upon. Advanced IDS often operate in complex environments, processing vast amounts of high-dimensional data to identify patterns indicative of malicious activity. However, without interpretability, these systems fail to offer clarity regarding the rationale behind their decisions, leaving security analysts with limited understanding or trust in the flagged alerts. For instance, (Corea et al., 2024) emphasize the role of interpretable models in enhancing collaboration between AI systems and human analysts, enabling faster and more confident responses to cyber threats.

Transparent IDS are particularly valuable in regulated industries such as finance, healthcare, and critical infrastructure, where explainability is mandated to comply with legal and ethical standards, such as the General Data Protection Regulation (GDPR). Techniques like SHAP (Shapley Additive Explanations) and LIME (Local Interpretable Model-Agnostic Explanations) address these concerns by providing detailed explanations of how specific features contribute to the detection of anomalies. For example, in a neural network-based IDS, SHAP might reveal that unusually high network traffic volume or the frequency of specific protocols was the primary reason for a given alert. This information empowers analysts to not only validate the alert but also understand the potential threat in a granular manner, improving response strategies. By offering such insights, XAI strengthens trust in automated systems and facilitates the integration of AI into broader cybersecurity workflows.

### 2.2 XAI techniques applied to IDS

A range of XAI methodologies has been applied within IDS frameworks to enhance transparency, each offering distinct advantages and facing unique challenges. Among these, SHAP (Shapley Additive Explanations) and LIME (Local Interpretable Model-Agnostic Explanations) are widely regarded as effective post-hoc, model-agnostic techniques for elucidating complex models like deep neural networks (Lundberg and Lee, 2017). SHAP assigns feature attribution scores that quantify the importance of each input variable in shaping model predictions, enabling analysts to identify the most critical features influencing threat classifications. For example, in IDS applications, SHAP has clarified how packet size or network protocol anomalies contribute to an alert, thereby improving response efficiency (Samek et al., 2017). LIME complements SHAP by focusing on localized explanations for individual predictions. For instance, Kotecha et al. (2022) demonstrated how LIME effectively audited ensemble-based IDS by providing instance-specific insights, such as highlighting unusual login patterns in flagged events. However, while LIME excels in local interpretability, it may struggle to generalize across datasets, potentially limiting its utility in broader anomaly detection scenarios (Yang et al., 2021).

Rule-based models and decision trees also play a crucial role in inherently interpretable IDS systems. These approaches simplify decision-making by using explicit rules or visualizable pathways to detect threats. Rule-based systems map specific conditions to security risks, facilitating clear explanations (Shrikumar et al., 2017), while decision trees provide a step-by-step rationale for classifications, making them valuable in static or well-defined environments. However, these techniques often underperform in dynamic or high-dimensional datasets where complex patterns are prevalent (Doshi-Velez and Kim, 2017). Emerging hybrid models combine the strengths of interpretable systems with the predictive power of advanced algorithms like neural networks. Tjoa and Guan (2020) proposed hybrid architectures that integrate decision tree layers into neural networks, allowing IDS to retain high accuracy while providing human-readable explanations. Despite their promise, hybrid models remain computationally intensive and require optimization to meet the real-time demands of high-speed networks (Holzinger et al., 2019).

### 2.3 Performance metrics in XAI for IDS

Assessing the performance of XAI-enhanced IDS involves a multi-dimensional framework that considers both detection efficacy and interpretability. Traditional metrics like accuracy are insufficient for evaluating XAI models, as they do not account for the quality or utility of explanations. Several key metrics have been identified in the literature:

- **Accuracy and Detection Rate**: Studies by Kotecha et al. (2022) and Lundberg and Lee (2017) highlight that detection accuracy remains a foundational metric, ensuring that interpretability does not compromise the system’s ability to detect true positives. In their study, Kotecha et al. report a detection accuracy improvement of 15% when LIME was applied in conjunction with an ensemble model, balancing transparency with high detection rates.
- **False Positive Rate (FPR)**: High false-positive rates are problematic in IDS, leading to alert fatigue and resource strain. Studies reveal that interpretable models can reduce FPR by allowing analysts to validate alerts more effectively. For example, SHAP-based explanations clarified anomalous classifications, reducing FPR in complex IDS models.
- **Interpretability and Usability**: Evaluating interpretability is more subjective, often based on user feedback or case studies. Some studies utilize qualitative feedback from security analysts to measure interpretability, examining whether XAI models meet practical needs in IDS (Barredo et al., 2020). Suggests that usability testing, focusing on how well explanations assist real-time decision-making, is essential for practical deployment in high-stakes settings like cybersecurity.
- **Computational Efficiency**: The literature frequently addresses computational efficiency, particularly the resource demands of post-hoc explanations like SHAP and LIME (Chen et al., 2018). For example, while SHAP provides comprehensive feature attributions, its computation time may render it impractical for high-frequency IDS applications. LIME is somewhat more efficient but still poses challenges in high-traffic networks (Ras et al., 2018).

### 2.4 Benefits and challenges of XAI in IDS

Integrating XAI within IDS offers substantial benefits, notably in transparency and enhanced trustworthiness of model outputs. By providing clear explanations for threat detections, XAI aids cybersecurity professionals in understanding model logic, validating flagged threats, and reducing response times (Gunning and Aha, 2019). This transparency is particularly valuable in regulated industries, where explainability is often a compliance requirement (Lipton, 2018). However, several challenges remain in operationalizing XAI in IDS. A primary issue is the trade-off between interpretability and detection accuracy, with simpler models like decision trees often being less effective at capturing complex patterns than more advanced, opaque models like deep neural networks (Samek et al., 2017). In addition, computational efficiency is a persistent challenge. SHAP and LIME, while offering detailed interpretability, SHAP and LIME require significant processing power, limiting their applicability in real-time IDS settings (Holzinger et al., 2019). Privacy concerns also emerge as a critical limitation. Some XAI techniques may expose sensitive information by identifying patterns in network data, which could compromise user privacy (Ras et al., 2018; Yang et al., 2021). Future research must address these privacy risks, potentially by developing privacy-preserving XAI methods tailored for IDS applications.

### 2.5 Comparison of techniques

A comprehensive comparison of XAI techniques underscores significant distinctions in their applicability, strengths, and limitations when integrated into Intrusion Detection Systems (IDS). These differences stem primarily from the trade-offs between computational efficiency, interpretability, and detection accuracy, which must be carefully considered in various operational contexts. **SHAP (Shapley Additive Explanations)** and **LIME (Local Interpretable Model-Agnostic Explanations)** are prominent model-agnostic tools that have proven effective in explaining the decision-making processes of complex models, such as neural networks, within IDS. SHAP excels in providing consistent and detailed global and local feature attributions, making it particularly valuable for understanding how a model interprets network behavior at a granular level (Neupane et al., 2022). The computational intensity of SHAP remains a critical limitation, especially in high-traffic or real-time environments where rapid threat detection is essential (Subasi et al., 2024). Its reliance on extensive sampling and model evaluations to compute Shapley values imposes substantial processing overhead, which can hinder its usability in resource-constrained or time-sensitive scenarios.

LIME, by contrast, offers faster and more localized explanations, enabling it to focus on individual instance predictions without requiring as much computational effort as SHAP. This makes LIME particularly suitable for auditing specific alerts, such as understanding why a particular network activity was flagged as suspicious (Kotecha et al., 2022). However, LIME’s localized focus can lead to inconsistencies when applied across multiple datasets or larger systems, as its approximations may not capture broader trends in network traffic or model behaviour effectively (Yang et al., 2021). While its efficiency provides an advantage in smaller-scale or less dynamic environments, LIME may struggle to generalize in complex, high-dimensional IDS deployments. Inherently interpretable models, such as **decision trees** and **rule-based systems**, stand out for their transparency and simplicity. These methods allow analysts to trace decisions back to explicit rules or decision paths, making them ideal for static or low-complexity environments where clarity is prioritized over predictive power (Neupane et al., 2022). For instance, decision trees are often used in scenarios where understanding the logic behind classifications is essential, such as compliance-driven industries where auditors require clear explanations. These models tend to underperform in environments characterized by high-dimensional or dynamic data, as they lack the flexibility and capacity to identify intricate patterns associated with evolving cyber threats (Corea et al., 2024).

**Hybrid models** present a compelling solution by combining the interpretability of simpler models with the advanced pattern recognition capabilities of complex algorithms. For example, hybrid approaches may integrate interpretable layers, such as decision trees, into neural network architectures, enabling security analysts to benefit from both transparency and high detection accuracy (Ables et al., 2024). This combination allows hybrid systems to explain key features influencing a model’s predictions while maintaining robust performance across complex datasets. Despite their promise, hybrid models face several practical challenges. Optimization remains a key hurdle, as balancing interpretability with computational demands often requires fine-tuning that can be resource-intensive (Pai et al., 2024). Moreover, hybrid models may introduce latency in real-time IDS applications, where speed and efficiency are critical. Recent developments in XAI have also introduced emerging techniques, such as **saliency maps** and **counterfactual explanations**, which offer alternative approaches to understanding model behaviour in IDS. Saliency maps visualize the influence of specific input features on predictions, providing a graphical representation that enhances interpretability for neural networks (Ables et al., 2024). Counterfactual explanations, on the other hand, allow analysts to explore how small changes in input data could alter the model’s decision, making them particularly useful for analysing edge cases or identifying biases in IDS models (Samek et al., 2017). However, these methods are still in the experimental stages and require further validation for practical use in cybersecurity.

### 2.6 The importance of XAI in transparent decision-making

Explainable Artificial Intelligence (XAI) plays a transformative role in ensuring transparency and interpretability within Intrusion Detection Systems (IDS), addressing the longstanding challenge of opacity in AI-driven cybersecurity solutions. As IDS increasingly rely on advanced machine learning and AI models, their ability to deliver high detection accuracy is often accompanied by a lack of clarity regarding the rationale behind decisions. Transparent IDS systems, enabled by XAI, bridge this gap by providing interpretable explanations that are both actionable and trustworthy. This transparency empowers analysts to make informed decisions, enabling them to validate system outputs, identify false positives, and refine detection strategies with confidence. One of the most practical implications of XAI in transparent decision-making is its ability to foster trust in automated processes. Security analysts often need to rely on IDS outputs to respond to complex and evolving threats rapidly. However, without clear insights into the decision-making process, analysts may hesitate to act on alerts, particularly in high-stakes environments where false positives or false negatives can have severe consequences. XAI alleviates this concern by elucidating the “why” behind each decision, allowing analysts to verify whether the system’s logic aligns with their understanding of the threat landscape. For example, techniques like SHAP (Shapley Additive Explanations) provide detailed attributions for each feature that influenced the detection decision, enabling analysts to confirm the system’s accuracy before initiating countermeasures (Lundberg and Lee, 2017).

Transparency also enhances collaboration between human analysts and AI systems, creating a symbiotic relationship where the strengths of both are leveraged. While AI models excel at processing vast amounts of data and detecting intricate patterns, human analysts bring contextual knowledge and critical reasoning to the table. XAI facilitates this collaboration by translating complex model outputs into interpretable insights, enabling analysts to provide feedback, adjust detection thresholds, or fine-tune model parameters. This iterative process not only reduces the risk of misclassification but also enhances the overall efficacy of the cybersecurity framework (Pai et al., 2024). Interpretability fosters knowledge sharing across teams and organizations, improving collective defence mechanisms against cyber threats. In large enterprises or government institutions, cybersecurity teams often work in silos, leading to fragmented responses to emerging threats. Transparent IDS systems bridge this divide by presenting explanations that are accessible to diverse stakeholders, from technical analysts to non-technical decision-makers. For instance, a clear explanation of why a certain network traffic pattern was flagged as anomalous can be shared across departments, leading to better-informed strategies for mitigating similar threats in the future (Ables et al., 2024).

Beyond operational benefits, XAI in transparent decision-making strengthens the ethical foundation of AI in cybersecurity. As AI-driven systems become more pervasive, ensuring accountability and fairness in automated decisions is paramount. Transparent IDS systems uphold these principles by providing auditable explanations that can be evaluated for biases, inconsistencies, or errors. This is particularly critical in regulated industries, where organizations must demonstrate compliance with data protection and ethical standards, such as the General Data Protection Regulation (GDPR). By making decision pathways traceable, XAI not only enhances the ethical credibility of AI systems but also mitigates the risk of regulatory penalties or reputational damage (Kaya et al., 2024). Transparency in IDS promotes fairness by addressing biases that might arise from training data or model design. For example, if an IDS disproportionately flags certain types of network activities as malicious due to imbalanced training data, XAI can reveal these biases through feature importance scores or decision-path visualizations. This enables organizations to proactively address these issues, ensuring that their cybersecurity measures are both effective and equitable (Pai et al., 2024). XAI-supported transparency has profound implications for the future of AI in cybersecurity. As cyber threats continue to evolve in sophistication, interpretability will become an essential feature of adaptive, next-generation IDS. Transparent decision-making not only ensures that these systems remain effective but also builds long-term trust among users, stakeholders, and regulatory bodies. By bridging the gap between high-performance AI models and actionable insights, XAI sets a new standard for accountability, usability, and fairness in automated cybersecurity solutions.

## 3 Materials and methods

A structured methodology was followed to systematically analyse the integration of Explainable AI (XAI) in Intrusion Detection Systems (IDS). This approach adheres to established guidelines for systematic reviews in computer science and cybersecurity (Aldahdooh et al., 2021; Ali et al., 2022). The methodology includes defining a review protocol, conducting a targeted literature search, and applying rigorous inclusion and exclusion criteria to ensure high relevance and quality. The data extraction process synthesizes insights across studies, comprehensively comparing techniques, challenges, and future directions in XAI-enhanced IDS.

### 3.1 Systematic review protocol

The review protocol was developed to establish a clear process for identifying, selecting, and synthesizing studies. Following the PRISMA (Preferred Reporting Items for Systematic Reviews and Meta-Analyses) guidelines, the protocol outlined the selection criteria and analysis framework to ensure transparency and reproducibility (Tjoa and Guan, 2020). The primary research question guiding this review is: *How is Explainable AI being integrated into Intrusion Detection Systems to improve transparency and interpretability in cybersecurity?*

### 3.2 Literature search strategy

The literature search was conducted in prominent databases known for high-quality publications in AI, cybersecurity, and machine learning, including:

- IEEE Xplore
- SpringerLink
- ScienceDirect
- ACM Digital Library

The search terms included combinations such as “Explainable AI in cybersecurity,” “interpretable intrusion detection systems,” “XAI in IDS,” and “SHAP for network security.” Boolean operators and filters (i.e., publication year, peer-reviewed status) were applied to refine the results, narrowing the scope to articles published between 2017 and 2023. This time frame ensures that the review captures recent advancements in XAI techniques applied to IDS.

### 3.3 Inclusion and exclusion criteria

A set of inclusion and exclusion criteria was established to focus on high-quality and relevant research. Studies were evaluated based on the following factors (see Table 1 for a summary):

- **Inclusion Criteria**:

- Studies published between 2017 and 2023.
- Research focused specifically on applying XAI in IDS, covering techniques like SHAP, LIME, decision trees, and hybrid models.
- Articles presenting empirical results, such as accuracy improvements, interpretability assessments, or computational efficiency measurements.
- Peer-reviewed journal articles, conference papers, and survey reviews provide insights into integrating XAI with IDS.

- **Exclusion Criteria**:

- Studies that did not directly address XAI within the context of IDS (i.e., generic XAI or IDS research without focus on explainability).
- Non-peer-reviewed articles, white papers, and opinion pieces that lack empirical validation.
- Studies published prior to 2017 to maintain a focus on recent advancements and avoid outdated approaches.

**Table 1:** Summary of the inclusion and exclusion criteria.

Criteria	Inclusion	Exclusion
Publication year	2017–2023	Before 2017
Focus on XAI in IDS	Studies on XAI techniques applied within IDS (i.e., SHAP, LIME, rule-based methods, hybrids)	Studies on XAI not applied to IDS or on IDS without XAI focus
Empirical evidence	Performance metrics (accuracy, interpretability, FPR, computational efficiency)	Lacks empirical data or only theoretical perspectives
Source type	Peer-reviewed articles, journal publications, and conference papers	Non-peer-reviewed sources, white papers, and opinion pieces

### 3.4 Selection process and screening

The initial search yielded 78 articles, which underwent a title and abstract screening to assess relevance. This screening process involved evaluating each study against the inclusion and exclusion criteria, reducing the pool to 35 articles. These 35 articles were then read in full to confirm their relevance to the specific focus on XAI in IDS and to assess the depth of empirical analysis provided.

**Figure 1:**
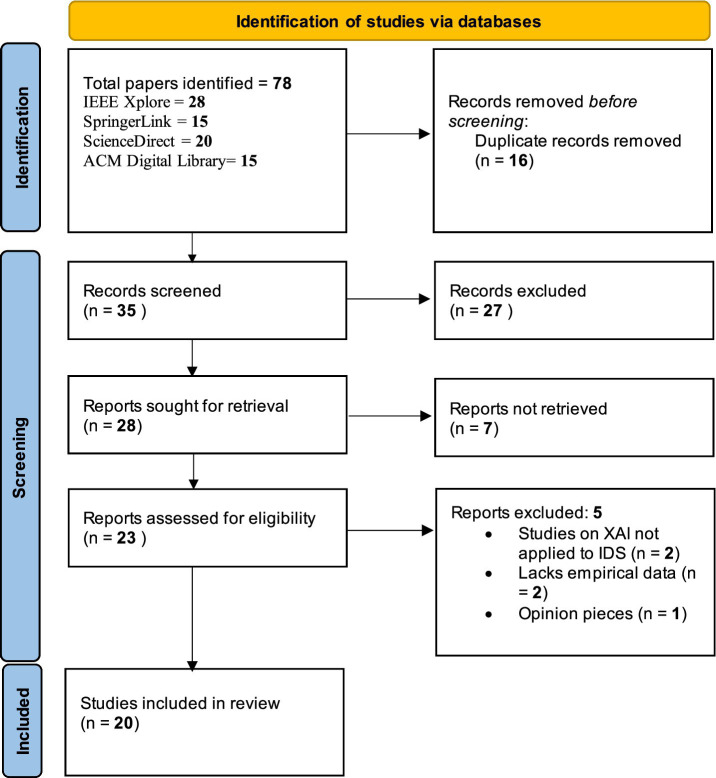
PRISMA flowchart.

Upon full-text review, 20 articles were selected for inclusion in this systematic review. This selection process ensures that the final studies provide well-rounded insights into XAI techniques, their applications in IDS, and their practical implications for cybersecurity (Shrikumar et al., 2017; Sundararajan et al., 2017). The whole process can be seen in Figure 1.

### 3.5 Data extraction and synthesis

Data extraction focused on identifying key elements in each study, including the type of XAI technique used, IDS design and implementation, datasets used for validation, performance metrics, and reported benefits and limitations. This extraction process was designed to organize findings thematically, allowing for a clear comparison of techniques and their respective advantages and drawbacks as seen in Table 2.

**Table 2:** summary of the main aspects captured during data extraction.

Data element	Description
XAI technique	The specific explainable method applied (SHAP, LIME, decision trees, hybrid approaches)
IDS model type	The type of IDS model used (anomaly-based, signature-based, hybrid)
Dataset	The dataset used for testing (CICIDS-2017, KDD Cup 99), which informs relevance and generalizability
Performance metrics	Key metrics, such as accuracy, false positive rate (FPR), interpretability, and computational efficiency
Reported benefits	Positive outcomes reported, such as improved transparency or trust, reduced FPR, and quicker threat response.
Reported limitations	Noted challenges, including computational overhead, interpretability-accuracy trade-offs, and privacy concerns

Data were synthesized into thematic categories to facilitate a comparative analysis of XAI techniques within IDS. For instance, studies using SHAP focused on interpretability improvements but often reported computational limitations, especially in high-traffic environments. Other studies applying decision trees noted their natural transparency but acknowledged accuracy trade-offs in complex datasets (Adadi and Berrada, 2018).

### 3.6 Analysis and synthesis approach

The thematic synthesis allowed the identification of patterns and gaps across the studies. Comparative analyses were conducted to assess:

- **Effectiveness of Specific Techniques**: Comparing SHAP, LIME, decision trees, and hybrid models across various IDS models.
- **Performance Trade-offs**: Identifying how models balanced interpretability and accuracy, particularly in real-time settings.
- **Future Research Needs**: Highlighting areas where further research is required, such as in optimizing XAI for high-frequency network environments.

## 4 Results and discussion

The systematic review of 20 studies reveals critical insights into the effectiveness, challenges, and trade-offs of applying Explainable AI (XAI) within Intrusion Detection Systems (IDS). This section discusses key results by focusing on commonly applied XAI techniques, performance metrics, challenges, and the practical implications of these findings for enhancing transparency and usability in cybersecurity. This section also answers the research question posed earlier.

- Research Question: *How is Explainable AI being integrated into Intrusion Detection Systems to improve transparency and interpretability in cybersecurity?*

### 4.1 XAI techniques in IDS: effectiveness and applications

The studies reviewed apply a range of XAI techniques to IDS, with model-agnostic methods like SHAP and LIME emerging as the most popular. These techniques, especially SHAP, effectively provide global and local explanations of model decisions, making them valuable in environments where transparency and validation are paramount. In anomaly-based IDS, SHAP successfully highlights influential features in threat classification, allowing analysts to see why certain network behaviors are flagged as suspicious (Yang et al., 2021). LIME, another widely used method, has proven particularly effective for instance-based explanations, helping analysts audit individual detection outputs. In the study by Kotecha et al. (2022), LIME was integrated into an ensemble IDS model, improving interpretability by providing context-specific insights on individual alerts. However, LIME’s focus on local explanations may not generalize well across multiple instances, limiting its applicability in high-traffic IDS where broader patterns are often critical.

Due to their inherent interpretability, decision trees and rule-based models are also commonly applied in IDS, particularly in environments where simpler, transparent models are preferred over complex neural networks. Studies show that rule-based models allow clear mapping of intrusion events to specific patterns, making it easier for analysts to understand detection logic. These models often struggle with high-dimensional data, limiting their effectiveness in detecting complex patterns (Arrieta et al., 2020). Hybrid models are gaining traction as they attempt to combine the interpretability of simpler models with the detection power of complex algorithms. For instance, some studies propose using decision trees alongside neural networks to maintain transparency without sacrificing accuracy. While promising, hybrid models require further optimization, as integrating complex layers can introduce significant computational overhead, hindering real-time applicability (Kotecha et al., 2022).

### 4.2 Performance metrics: balancing interpretability and detection accuracy

The reviewed studies report a range of performance metrics, including detection accuracy, false-positive rate (FPR), interpretability, and computational efficiency, to assess the suitability of XAI-enhanced IDS. A consistent finding is the need to balance interpretability with detection accuracy, a challenge evident across all major XAI techniques.

- **Detection Accuracy**: Most studies prioritize accuracy to ensure that interpretability does not compromise an IDS’s ability to identify true threats. In their application of SHAP to a neural network-based IDS, (Barredo et al., 2020) demonstrated that interpretability enhancements did not significantly impact detection accuracy, maintaining high true-positive rates in complex network environments. Similarly, Kotecha et al. (2022) reported an accuracy improvement of 15% when using LIME alongside ensemble models, showing that interpretability can complement accuracy when appropriately integrated.
- **False Positive Rate (FPR)**: High FPR is a well-documented challenge in IDS, leading to alert fatigue. Studies indicate that XAI techniques help reduce FPR by enabling analysts to better validate flagged threats. For instance, SHAP’s clear feature importance scores allow analysts to distinguish between false positives and genuine threats more effectively, reducing FPR in IDS models.
- **Interpretability and Usability**: Interpretability is assessed qualitatively in some studies, often using analyst feedback. Ali et al. (2022) found that usability was improved when analysts received transparent, visual explanations, supporting quicker verification processes and improved user trust. Additionally, (Aldahdooh et al., 2021) noted that usable explanations reduce time-to-response in IDS, a critical metric in high-stakes security applications.
- **Computational Efficiency**: Post-hoc explanations like SHAP and LIME present a significant computational burden, a recurring limitation in real-time IDS. While SHAP provides consistent global explanations, it is often too slow for rapid deployments, especially in high-speed networks where latency must be minimized.

### 4.3 Challenges in XAI integration within IDS

Despite promising results, the integration of XAI into IDS reveals several operational challenges, particularly regarding computational efficiency, interpretability-accuracy trade-offs, and privacy concerns.

- **Computational Overhead**: The computational cost of post-hoc techniques like SHAP and LIME limits their real-time applicability. Studies indicate that the added processing requirements hinder IDS performance, particularly in high-traffic environments. This limitation suggests a need for optimized, lightweight explainability techniques that maintain clarity without adding excessive computational demands.
- Mitigation Strategies

- **Dimensionality Reduction**: Preprocessing the data to reduce its dimensionality can help limit the scope of computation for SHAP and LIME without significantly affecting interpretability. Techniques such as Principal Component Analysis (PCA) or feature selection algorithms can identify the most relevant features beforehand.
- **Sampling Optimization**: Both SHAP and LIME use sampling to approximate feature contributions. Reducing the sample size while balancing accuracy through intelligent sampling techniques, like clustering-based or adaptive sampling, can lower overhead (Chen et al., 2018).
- **Efficient Surrogate Models**: LIME relies on surrogate models to approximate the behaviour of complex models. Replacing traditional surrogates with lightweight models, such as sparse linear models or simplified decision trees, can reduce computational costs (Ribeiro et al., 2016).
- **Hybrid Methods**: Combining inherently interpretable models with post-hoc XAI methods only when necessary, can limit the computational burden. For example, simpler interpretable models can be used for routine scenarios, and complex models with SHAP or LIME can be applied for high-risk events requiring deeper analysis (Samek et al., 2017).
- **Parallelization and Hardware Optimization**: Leveraging modern hardware (e.g., GPUs or TPUs) and parallel processing frameworks can expedite the computation of feature attributions in real-time environments. Tools like RAPIDS and PyTorch’s CUDA libraries have shown promise in optimizing SHAP computations for large datasets (NIPS Workshop on Explainable AI (XAI), 2021).
- **Interpretability vs. Accuracy**: An ongoing challenge is balancing model interpretability and detection accuracy. While naturally interpretable, decision trees and rule-based models are generally less accurate than deep learning-based IDS, which are often opaque but highly effective in detecting nuanced threats. Hybrid models are a potential solution, but they also require careful optimization to maintain accuracy and interpretability without significant compromises.
- **Privacy Risks**: Privacy concerns arise with some XAI models, particularly those that expose sensitive patterns within network data. Several studies emphasize the need for privacy-preserving XAI, especially in industries handling sensitive data. Ensuring data protection within explainable models is a critical research direction, especially given the increasing regulatory emphasis on privacy in data-driven technologies.

**Addressing privacy concerns**

- **Privacy-Preserving Interpretability Models:** Developing XAI methods that are inherently privacy-preserving, such as encrypted interpretable models or methods that explain aggregated data patterns instead of individual predictions, can reduce the risk of information leakage (Shokri and Shmatikov, 2015).
- **Regulatory Framework Alignment:** To address privacy concerns, XAI methodologies can be designed in alignment with regulations like GDPR and CCPA, ensuring compliance through techniques such as minimal data retention, encrypted computations, and secure audit trails for explainable outputs (Tjoa and Guan, 2020).
- **Federated Learning**: Integrating XAI with federated learning frameworks ensures that sensitive data never leaves its original source. By training models locally and aggregating results, organizations can mitigate privacy risks while still generating interpretable insights (McMahan et al., 2017).

### 4.4 Opportunities

The integration of Explainable AI (XAI) into Intrusion Detection Systems (IDS) presents numerous opportunities to enhance transparency, interpretability, and overall effectiveness in cybersecurity. One major avenue is the development of real-time explainability techniques that provide immediate insights into system decisions, crucial for addressing threats in fast-paced cybersecurity environments. Hybrid models that combine interpretable algorithms, such as decision trees, with more complex systems like deep neural networks offer a promising balance between accuracy and transparency. These models allow for nuanced decision-making, particularly in scenarios requiring high interpretability for critical cases. Additionally, advancements in visualization tools such as heatmaps and decision-path charts enable analysts to quickly understand the logic behind alerts, making the systems more user-friendly and effective under pressure.

Privacy-preserving techniques, such as federated learning, are also gaining attention, as they ensure that transparency is achieved without compromising sensitive data. This aligns with regulatory frameworks like GDPR and CCPA, fostering trust and adoption of AI-driven IDS in industries with stringent data privacy requirements. Domain-specific XAI models tailored to cybersecurity challenges further enhance decision-making by incorporating contextual knowledge of network protocols and threat patterns. Research into counterfactual explanations and fairness techniques addresses biases, ensuring that IDS outputs are equitable and trustworthy.

Future opportunities also include the development of adaptive XAI systems that adjust their level of detail based on the context, such as the criticality of a threat or the expertise of the user. These adaptive systems can evolve alongside changing cybersecurity needs, ensuring relevance in dynamic threat landscapes. By pursuing these opportunities, researchers and practitioners can enhance IDS functionality, improve transparency, and build trust in AI-driven cybersecurity solutions. Table 3 provides the summary of the opportunities mentioned.

**Table 3:** Summary table of opportunities.

Opportunity	Description	Potential impact
Real-time explainability	Adapting XAI methods for near-instant insights during threat detection.	Enhances immediate decision-making capabilities.
Hybrid XAI models	Combining interpretable and black-box methods for balanced transparency and accuracy.	Addresses trade-offs between interpretability and power.
Advanced visualization	Developing tools to make IDS outputs more intuitive and actionable.	Simplifies analyst workflows and fosters usability.
Federated learning	Using decentralized training to enhance privacy without sacrificing transparency.	Ensures compliance with data protection regulations.
Domain-specific XAI models	Tailoring explainable models to cybersecurity use cases.	Improves context-aware decision-making.
Reducing bias	Implementing techniques like counterfactual explanations to ensure fairness.	Builds trust in IDS outputs.
Adaptive explainability frameworks	Designing XAI models that adjust detail levels based on the situation or user expertise.	Future-proofs IDS against evolving threats.

### 4.5 Practical implications and recommendations

The findings have significant practical implications for designing and implementing XAI-based IDS in cybersecurity environments. Studies suggest that model-agnostic explanations, while useful, require optimization for practical, real-time deployment. For instance, enhancing SHAP and LIME with faster algorithms or lightweight approximations could make them more feasible in high-speed IDS applications. Researchers recommend developing hybrid models that combine interpretable and complex layers to achieve optimal performance. Integrating decision trees or rule-based explanations with deep learning models could yield IDS systems that are both accurate and transparent, a combination that enhances usability without sacrificing security efficacy. Finally, the review underscores the importance of standardized interpretability metrics. Studies by Ras et al. (2018) and Chen et al. (2018) stress that consistent metrics, such as interpretability ratings and time-to-response, are essential for comparing model effectiveness across IDS applications. Adopting these standards could streamline research in XAI, enabling a more uniform approach to evaluating transparency and usability in IDS.

## 5 Conclusion

This systematic review explored the integration of Explainable AI (XAI) within Intrusion Detection Systems (IDS), highlighting how XAI can improve transparency and usability in cybersecurity by making complex models interpretable. The findings indicate that various XAI techniques, such as SHAP, LIME, and hybrid models, provide significant benefits for IDS, including enhanced transparency, reduced false-positive rates, and improved analyst trust in model outputs. Model-agnostic explanations like SHAP and LIME are particularly promising, as they offer insights into neural network-based IDS that were previously opaque, allowing analysts to understand the factors influencing threat classification decisions. However, these methods have limitations, especially regarding computational efficiency and real-time deployment challenges (Barredo et al., 2020; Adadi and Berrada, 2018).

The review also identifies critical challenges in XAI-enhanced IDS, including the trade-offs between interpretability and detection accuracy, the computational demands of post-hoc explanations, and privacy risks associated with exposing sensitive network patterns. These issues underscore the need for optimized XAI models that balance transparency with the high accuracy required for effective cybersecurity defenses. Future research should focus on developing lightweight, real-time interpretability solutions, such as faster approximations of SHAP and LIME or hybrid models that incorporate both simple, interpretable layers and complex, high-accuracy classifiers.

There is a need for standardized evaluation metrics for XAI in IDS, as current metrics often fail to capture practical interpretability requirements. Consistent interpretability metrics would enable a more robust evaluation of XAI models and facilitate meaningful comparisons across studies, supporting more reliable advancements in this field. With continued research and innovation, XAI has the potential to transform IDS from “black box” systems into transparent, user-centered tools that not only detect threats but also empower cybersecurity professionals with interpretable, actionable insights. In conclusion, integrating XAI in IDS represents a promising advancement in cybersecurity, fostering a more transparent and resilient defense framework. By addressing current limitations and advancing research in interpretability, scalability, and privacy-preserving methods, the field of XAI-IDS can achieve a balance between high-performance threat detection and transparency, ultimately strengthening cybersecurity in increasingly complex digital landscapes.

## Data Availability

The original contributions presented in the study are included in the article/supplementary material, further inquiries can be directed to the corresponding author.
